# Implicit associations between individual properties of color and sound

**DOI:** 10.3758/s13414-018-01639-7

**Published:** 2018-12-13

**Authors:** Andrey Anikin, N. Johansson

**Affiliations:** 1 Division of Cognitive Science, Department of Philosophy, Lund University, Box 192, SE-221 00 Lund, Sweden; 2 Center for Language and Literature, Lund University, Lund, Sweden

**Keywords:** Cross-modal correspondences, Color, Synesthesia, Sound symbolism, Implicit associations test

## Abstract

We report a series of 22 experiments in which the implicit associations test (IAT) was used to investigate cross-modal correspondences between visual (luminance, hue [R-G, B-Y], saturation) and acoustic (loudness, pitch, formants [F1, F2], spectral centroid, trill) dimensions. Colors were sampled from the perceptually accurate *CIE-Lab* space, and the complex, vowel-like sounds were created with a formant synthesizer capable of separately manipulating individual acoustic properties. In line with previous reports, the loudness and pitch of acoustic stimuli were associated with both luminance and saturation of the presented colors. However, pitch was associated specifically with color lightness, whereas loudness mapped onto greater visual saliency. Manipulating the spectrum of sounds without modifying their pitch showed that an upward shift of spectral energy was associated with the same visual features (higher luminance and saturation) as higher pitch. In contrast, changing formant frequencies of synthetic vowels while minimizing the accompanying shifts in spectral centroid failed to reveal cross-modal correspondences with color. This may indicate that the commonly reported associations between vowels and colors are mediated by differences in the overall balance of low- and high-frequency energy in the spectrum rather than by vowel identity as such. Surprisingly, the hue of colors with the same luminance and saturation was not associated with any of the tested acoustic features, except for a weak preference to match higher pitch with blue (vs. yellow). We discuss these findings in the context of previous research and consider their implications for sound symbolism in world languages.

## Introduction

People have long been curious about why certain sounds and colors somehow “match.” Hearing a particular sound automatically and consistently produces a conscious experience of a particular color (Ward, 2013) in people with sound-color synesthesia. Non-synesthetes also often have strong intuitions about which sounds and colors go well together. It is a matter of ongoing debate to what extent such cross-modal correspondences share mechanisms with synesthesia (e.g., Lacey, Martinez, McCormick, & Sathian, 2016; Spence, 2011), but they certainly affect both perception and the way we talk about the world. For example, it seems natural to refer to high-frequency sounds as “bright,” although there is no *a priori* reason to associate visual brightness with auditory frequency. The pervasiveness of such metaphors emphasizes the importance of cross-modal correspondences not only for human perception but for language as well (Bankieris & Simner, 2015; Ramachandran & Hubbard, 2001; Sidhu & Pexman, 2018). Iconicity, or the motivated association between sound and meaning, has deepened our understanding of how human language and cognition evolved, as well as of how language continues to evolve culturally, by exposing several mechanisms that influence word formation and sound change. The concepts affected by lexical iconicity, or *sound symbolism*, generally have functions that relate to description or perception. Coupled with extensive perceptual evidence of cross-modal sound-color associations, this makes the names of colors good candidates both for finding evidence of sound symbolism (Blasi, Wichmann, Hammarström, Stadler, & Christiansen, 2016; Johansson, Anikin, Carling, & Holmer, 2018) and for relating it to potential psychological causes.

In the present article we address the psychological component of this problem by looking at how different color properties such as luminance, saturation, and hue are mapped onto acoustic properties such as loudness, pitch, and spectral characteristics. We begin by reviewing the extensive, but methodologically diverse and sometimes contradictory previous literature on sound-color associations and then report the results of our own experiments, in which we attempted to systematically test for cross-modal correspondences between linguistically meaningful acoustic features and individual perceptual dimensions of color.

It has long been known that people map auditory loudness onto visual luminance both in explicit matching tasks (Marks, 1974; Root & Ross, 1965) and in tests for implicit associations (Marks, 1987). There is some controversy surrounding the exact nature of matched dimensions that we return to in the *Discussion*, but in general, luminance-loudness associations are a straightforward example of so-called prothetic cross-modal correspondences that are based on the amount rather than the quality of sensory experience in two modalities (Spence, 2011). Loud sounds and bright colors share the property of being high on their respective prothetic dimensions and are therefore grouped together.

Pitch – the property describing how “high” or “low” a tonal sound appears to be – is reliably associated with luminance (Hubbard, 1996; Marks, 1974; Mondloch & Maurer, 2004; Ward, Huckstep, & Tsakanikos, 2006) and perhaps also with saturation (Hamilton-Fletcher, Witzel, Reby, & Ward, 2017; Ward et al., 2006). Unlike loudness, pitch is usually considered a metathetic rather than a prothetic dimension (Spence, 2011), in the sense that higher pitch is not “larger” or “greater” than low pitch, but qualitatively different. As a result, it is normally assumed that pitch is mapped onto sensory dimensions in other modalities, such as luminance, based on some qualitative correspondence between them. One complication is that some of the reported associations between pitch and color (Table 1) may have been caused by accompanying changes in loudness. The sensitivity of human hearing is frequency-dependent, and within the commonly tested range of approximately 0.2–3 kHz the subjective loudness of pure tones with the same amplitude monotonically increases with frequency (Fastl & Zwicker, 2006). It is therefore not enough to use stimuli normalized for peak or root mean square amplitude – the sound with the higher pitch may still be subjectively experienced as louder, introducing a confound. However, there is some evidence that the association of pitch with luminance (Klapetek et al., 2012), saturation, and hue (Hamilton-Fletcher et al., 2017) appears to hold even when the subjective loudness is held constant, indicating that cross-modal correspondences involving pitch are not entirely mediated by loudness.

**Table 1:** Summary of previous reports of sound-color associations and our own data

Acoustic feature	Visual feature	Association	References	Our data	Proposed mechanism
Loudness	Luminance	Loudness ~ brightness	Bond & Stevens, 1969 Root & Ross, 1965	Loudness ~ darker gray on white background	Prothetic matching of loudness and visual salience
		Loudness ~ lightness (inconsistent, depending on background)	Marks, 1974, 1987		
	Hue	Loudness ~ orange/yellow (vs. blue)	Hamilton-Fletcher et al., 2017 Kim, Gejima, Iwamiya, & Takada, 2011 Menzel, Haufe, & Fastl, 2010	No association	Semantic matching^§^
		Loudness ~ red (vs. green)	Kim et al., 2011 Menzel et al., 2010		
	Saturation	Loudness ~ high saturation	Giannakis, 2001 Hamilton-Fletcher et al., 2017 Kim et al., 2011 Panek & Stevens, 1966	Loudness ~ high saturation	Prothetic matching of loudness and saturation
Pitch	Luminance	Pitch ~ luminance	Giannakis, 2001 Hubbard, 1996 Jonas, Spiller, & Hibbard, 2017 Klapetek, Ngo, & Spence, 2012 Ludwig, Adachi, & Matsuzawa, 2011 Marks, 1974, 1987 Martino & Marks, 1999 Melara, 1989 Mondloch & Maurer, 2004 Orlandatou, 2012 Ward et al., 2006 Watanabe, Greenberg, & Sagisaka, 2014	Pitch ~ lighter gray on white background	Metathetic matching of frequency and lightness
		No effect: pitch ~ visual contrast	Evans & Treisman, 2010		
	Hue	Pitch ~ yellow (vs. blue)	Hamilton-Fletcher et al., 2017 Hubbard, 1996 Orlandatou, 2012 Simpson, Quinn, & Ausubel, 1956	Pitch ~ blue (vs. yellow)	Semantic matching^§^
		No effect: pitch ~ blue (vs. red)	Bernstein & Edelstein, 1971		
	Saturation	Pitch ~ high saturation	Jonas et al., 2017 Ward et al., 2006	Pitch ~ high saturation	Prothetic matching of frequency and saturation^§^
Formants	Luminance	F1 ~ high luminance	Moos, Smith, Miller, & Simmons, 2014	No association	Metathetic matching of frequency and lightness
		F1 ~ low luminance	Kim, Nam, & Kim, 2017		
		F2 ~ high luminance	Kim et al., 2017 Moos et al., 2014		
		[i] [e] ~ bright colors [o] [u] ~ dark colors	Marks, 1975		
	Hue	F1 ~ red (vs. green)	Kim et al., 2017 Marks, 1975 Moos et al., 2014 Wrembel, 2009	No association	Semantic matching^§^
		F1 ~ blue (vs. yellow)	Kim et al., 2017		
		F2 ~ green (vs. red)	Moos et al., 2014 No effect in Kim et al., 2017		
		F2 ~ yellow (vs. blue)	Kim et al., 2017 Moos et al., 2014 Wrembel, 2009		
		High F2/F1 ratio ~ green vs. red	Marks, 1975		
	Saturation	F1 ~ saturation F2 ~ saturation	Jakobson, 1962 cited in Moos et al., 2014	-	§
Other	Luminance	-	-	Spectral centroid ~ lighter gray	Metathetic matching of frequency and lightness
				Trill ~ darker gray	Prothetic matching of visual and auditory saliency and/or metathetic matching of frequency and lightness
	Hue	Any power over 800 Hz ~ yellow (vs. blue)	Hamilton-Fletcher et al., 2017	Spectral centroid ~ blue (vs. yellow)^¶^	Semantic matching^§^
	Saturation	Spectral centroid ~ high saturation	Hamilton-Fletcher et al., 2017	Spectral centroid ~ high saturation	Prothetic matching of frequency and saturation^§^
		Noise vs. harmonic ~ low saturation	Orlandatou, 2012	-	§
				Trill ~ low saturation^¶^	Prothetic matching of visual and auditory saliency and/or metathetic matching of frequency and lightness

§ Uncertain mechanism

¶ Statistically marginal effect

Compared to the extensive research on color-loudness and color-pitch associations, there is less experimental evidence on how color is associated with spectral characteristics such as formants – frequency bands that are amplified by the vocal tract, creating different vowel sounds. In a large review of sound-color synesthesia spanning literally centuries of reports, Marks (1975, p. 308) concludes that certain vowels are reported to match different colors by synesthetes and non-synesthetes alike: [a] is associated with red and blue, [e] and [i] with yellow and white, [o] with red and black, and [u] with brown, blue, and black. More recent studies are largely consistent with Marks' summary (e.g., Miyahara, Koda, Sekiguchi, & Amemiya, 2012; Watanabe et al., 2014). The general rule appears to be that bright-sounding vowels, such as [i] and [e], are matched with bright colors, while dark-sounding vowels, such as [o] and [u], are matched with dark colors. The brightness of a vowel is sometimes said to be determined primarily by the second formant F2 (Marks, 1975), but in general raising the frequency of any formant tends to shift the balance of spectrum towards higher frequencies (Stevens, 2000). The center of gravity of a spectrum, also known as the spectral centroid, is a popular measure of the overall brightness or sharpness of musical timbre (Schubert, Wolfe, & Tarnopolsky, 2004), and an adjusted version of spectral centroid is used to approximate human ratings of sharpness in psychoacoustics (Fastl & Zwicker, 2006). Apparently, there is no direct evidence that the spectral centroid of complex tones with the same pitch is associated with visual luminance, but this effect is strongly predicted by the well-documented pitch-luminance associations and timbral consequences of raising the spectral centroid. There is also some experimental support for the idea that higher formants should be associated with greater luminance (Moos et al., 2014; but see Kim et al., 2017). An interesting unresolved issue is whether the association between formant frequencies and luminance is mediated by vowel quality or simply by the balance of low- and high-frequency energy in the spectrum. It seems intuitive that a vowel like [u] has an intrinsic “dark” quality that would not disappear by boosting high frequencies in the spectrum, but to the best of our knowledge, this assumption has not been tested.

There are also several reports linking formant frequencies to hue rather than luminance. Marks (1975) suggests that a high F2/F1 ratio is associated with green and a low F2/F1 ratio with red colors. Broadly consistent with this claim, Wrembel (2009) found that high front vowels, such as [i], were often matched with yellow or green hues. Furthermore, both synesthetes and non-synesthetes explicitly matched natural vowels with higher F1 to red rather than green in several experiments (Kim et al., 2017; Moos et al., 2014). Kim et al. (2017) report that yellow was associated with low F1 and high F2, although this relationship disappeared if they did not simultaneously vary the pitch of their synthetic vowels. Unfortunately, the presence of several confounds in most studies makes it difficult to determine what visual properties (hue, saturation, or luminance of the tested colors) were mapped to what acoustic properties (frequency of the first and second formants, F2/F1 ratio, or spectral centroid). In one of the most carefully controlled studies, Hamilton-Fletcher et al. (2017) discovered that the presence of energy above 800 Hz in the spectrum of complex synthetic tones was associated with yellow hues, even when participants were constrained to choose among equiluminant colors.

The key findings from the research on color-sound associations are presented in Table 1, with a particular emphasis on controlled experiments. Although by no means exhaustive, this summary highlights several contradictions and unresolved issues. Furthermore, many of the reported findings come from small studies with multiple potential confounds. In our opinion, the most significant progress in the field has been associated with three methodological advances:

1. *Controlling for visual confounds*. Until the last decade, researchers mainly worked with focal colors or approximations to the subjective color space, using contrasts such as light-dark or red-green. The recently pioneered use of perceptually accurate color spaces, such as *CIE-Luv* (Hamilton-Fletcher et al., 2017; Moos et al., 2014) and *CIE-Lab* (Kim et al., 2017), has the advantage of preserving subjective distances between colors while offering control over the separate dimensions of lightness, hue, and saturation. For example, there are several reports linking higher pitch to yellow (Orlandatou, 2012; Simpson et al., 1956). At the same time, focal yellow is also the brightest color (Witzel & Franklin, 2014), making it unclear whether yellow is associated with bright vowels because of its hue or because of its high luminance and saturation. By offering participants a choice among colors of the same luminance, Hamilton-Fletcher and co-workers (2017) demonstrated that yellow hues match higher frequencies in their own right, and not only because of their high luminance.
2. *Controlling for acoustic confounds*. Just as colors are defined by several perceptually distinct qualities, sounds have various acoustic properties that may contribute towards the discovered sound-color associations. The best-understood acoustic features are loudness and pitch, but speech-like harmonic sounds also vary in complex temporal and spectral characteristics such as formants, spectral noise, overall balance of low- and high-frequency energy in the spectrum, amplitude modulation, and so on. While loudness and pitch manipulations were already employed in early studies using synthetic white noise or pure tones (Marks, 1974; Root & Ross, 1965), modern techniques of formant synthesis enable researchers to create more naturalistic, speech-like sounds for testing. For example, Hamilton-Fletcher and co-workers (Hamilton-Fletcher et al., 2017) created a complex tone with several harmonics, the strength of which they could manipulate independently in order to change the spectral characteristics of their stimuli. Kim and co-authors (Kim et al., 2017) went a step further and used articulatory synthesis to manipulate formant frequencies in vowel-like sounds. This is potentially a highly promising approach, but at present a number of challenges remain. For example, raising F1 or F2 has the effect of also boosting all frequencies above them (Stevens, 2000). In addition, manipulations of pitch and spectral characteristics can have a major effect on the perceived loudness of the stimuli. This is usually ignored (with a few exceptions, e.g., Hamilton-Fletcher et al., 2017 and Klapetek et al., 2012), but in view of the strong association between loudness and luminance it is desirable to make sure that the contrasted sounds are experienced as equally loud.
3. *Testing for implicit associations*. Until the mid-twentieth century, all evidence on color-sound associations consisted of reports by individuals, often synesthetes, who explicitly matched sounds with colors (reviewed in Marks, 1975). This method of subjective matching remains dominant in the field, but it primarily taps into what Spence (2011) calls the “decisional level,” while it is also important to look for sound-color associations at a lower “perceptual level.” Explicit beliefs about which color matches which sound are presumably grounded in low-level sensory correspondences, but they can also be influenced by cultural factors and personal history. Just as psychologists use implicit measures in order to study socially undesirable prejudices and biases, researchers of cross-modal correspondences have employed the speeded classification task (Ludwig et al., 2011; Marks, 1987), cross-modal Stroop interference (Ward et al., 2006), the implicit associations test (IAT; Lacey et al., 2016; Miyahara et al., 2012; Parise & Spence, 2012), the “pip-and-pop effect” (Klapetek et al., 2012), and other alternatives to explicit matching. Subjects do not have to be aware of possessing certain cross-modal correspondences for them to be detected in implicit tasks, and the results are less likely to be affected by cultural norms or idiosyncratic personal preferences.

We designed our experimental task with these three methodological considerations in mind. Like Kim et al. (2017), we sampled colors from the *CIE-Lab* space and created synthetic vowels. However, we used an adapted version of the IAT (Parise & Spence, 2012) instead of explicit matching. As argued above, implicit measures are more suitable for addressing cross-modal correspondences at a lower sensory level, which arguably holds the key to color-sound associations. In addition, with the IAT we had full control over the visual and acoustic characteristics of the contrasted pairs of stimuli, thus avoiding many confounds that arise in matching studies. Our pairs of colors differed only on one dimension at a time: luminance, saturation, or hue. In contrast, hue and saturation typically co-vary in matching studies, even if luminance is held constant (as in Hamilton-Fletcher et al., 2017). As for the acoustic stimuli, our ambition was to combine the rich spectral structure of the synthetic vowels used by Kim et al. (2017) with the careful matching of acoustic features achieved by Hamilton-Fletcher et al. (2017). We used formant synthesis to create natural-sounding vowels and manipulated one acoustic feature at a time to create six contrasted pairs; we also performed a separate pilot study to ensure that all stimuli were comparable in terms of subjective loudness.

The principal disadvantage of the chosen design was that only two pairs of colors and sounds could be compared in a single IAT experiment. A large number of participants therefore had to be tested in order to explore multiple combinations of stimuli, and even then it was impractical to determine whether the relationship between two features, such as pitch and saturation, was linear or quadratic (cf. Ward et al., 2006), based on absolute or relative values of the associated features (cf. Hamilton-Fletcher et al., 2017), etc. Because of this methodological limitation, we focused only on detecting the existence of particular cross-modal correspondences, not on their shape or robustness to variation in visual and auditory stimuli. We therefore made both visual and auditory contrasts in our stimuli pairs relatively large, well above detection thresholds. We also opted to collect data online, which allowed us to recruit a large and diverse sample of participants rapidly and at a reasonable cost (Woods, Velasco, Levitan, Wan, & Spence, 2015). Our goal was to investigate systematically, and using exactly the same experimental task, many of the previously described color-sound associations summarized in Table 1. Because in many cases the existing evidence comes from methodologically diverse studies and includes potential confounds, we did not formulate formal hypotheses to be tested, but simply looked for evidence of sound-color associations across a broad range of visual and auditory contrasts.

## Methods

### Stimuli

Visual stimuli were squares of 800 × 800 pixels of uniform color shown on white background. Pairs of colors were chosen so as to differ along only one dimension in the *Lab* space: luminance (*L*), hue (green-red [*a*] or yellow-blue [*b*]), or saturation (*sat*). Saturation was defined as the Euclidean distance to the central axis of the *Lab* space corresponding to shades of gray (*a* = 0, *b* = 0). The visual stimuli did not necessarily correspond to focal colors, but they were different enough to be easily distinguishable (Table 2).

**Table 2:**
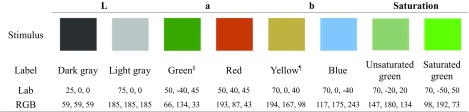
Contrasted pairs of visual stimuli

§ Due to a mistake, in one experiment (F2 – green/red contrast) the colors slightly differed in saturation: green was *Lab* [60, -40, 40] and red [60, 60, 40]

¶ Bright, focal yellow is much lighter than any bluish hue, so to keep luminance constant we had to oppose blue to a bronze-like, dark yellow

The investigated acoustic features were chiefly selected based on the strongest previously reported evidence of sound-color correspondences such as loudness, pitch, and spectrum. We also manipulated the frequencies of the first two formants, F1 and F2 – the two dimensions of the vowel chart – in order to connect the study more closely to natural speech sounds. In addition, the typologically most common trill, [r] (Mielke, 2004–2018; Moran, McCloy, & Wright, 2014), was also included due to its unique phonetic characteristics, such as its series of up to five pulses (Ladefoged & Maddieson, 1996, pp. 215–232), and because it has previously been found to be sound symbolically associated with the color green as well as words for movement and rotation (Johansson, Anikin, Carling, et al., 2018).

Acoustic stimuli were synthetic vowels created with *soundgen* 1.2.0*,* an open-source R package for parametric voice synthesis (Anikin, 2018). The voiced component lasted 350 ms, and the unvoiced component (aspiration) faded out over an additional 100 ms, so perceptually the duration was about 400 ms. The basic *soundgen* settings were shared by most stimuli and chosen so as to create a natural-sounding, gender-ambiguous voice pronouncing a short vowel. The fundamental frequency varied in a smooth rising-falling pattern between 160 and 200 Hz. Formant frequencies were equidistant, as in the neutral *schwa* [ə] sound (except when manipulated), and corresponded to a vocal tract length of 14 cm. Slight parallel formant transitions and aspiration were added to enhance the authenticity of stimuli. We opted to use diphthongs rather than static vowels for the contrasts that involved F1 or F2, so as to make the contrasts more salient. The manipulated formant moved up or down from a neutral *schwa* position, creating two different diphthongs.

As shown in Table 3, the spectral centroids of contrasted sounds with formant transitions were not exactly identical, but we did dynamically modify the strength of harmonics so as to achieve a relatively stable amount of high-frequency spectral energy and thereby mostly counteract the tendency for spectral centroid to shift in accordance with formant frequencies. In addition, to ensure that the subjectively experienced loudness of stimuli pairs would be as similar as possible (except when loudness was the tested contrast), the appropriate coefficients for adjusting the amplitude were estimated in a separate pilot study with five participants (Table 3, last column). All stimuli and R scripts for their generation can be downloaded from http://cogsci.se/publications.html together with the dataset and scripts for statistical analysis.

**Table 3:** Acoustic stimuli with the relevant soundgen settings

Manipulation	Contrast	Sound 1		Sound 2		Loudness equalization
		Key settings	Spectral centroid (Hz)	Key settings	Spectral centroid (Hz)	
Loudness	Two identical sounds, one 20 dB louder	Peak amplitude 0 dB	1,291	Peak amplitude -20 dB (1/10 of sound 1)	1,291	-
Pitch	Pitch difference of 1/2 octave	Low F0: 135-168-135 (-3 semitones)	1,252	High F0: 190-238-190 (+3 semitones)	1,242	-7.4 dB for low F0
F1	F1 either rises or falls 4 semitones from neutral	Rising F1: *formants = list(f1 = c(630, 790), f2 = 1900, f3 = 3160, f4 = 4430), rolloff = c(-8, -9)* ^§^	1,384	Falling F1]: *formants = list(f1 = c(630, 500), f2 = 1900, f3 = 3160, f4 = 4430), rolloff = c(-8, -7)* ^§^	1,463	-
F2	F2 either rises or falls 6 semitones from neutral	Rising F2: *formants = list(f1 = 630, f2 = c(1900, 2680), f3 = 3160, f4 = 4430), rolloff = c(-7.5, -9)* ^§^	1,659	Falling F2: *formants = list(f1 = 630, f2 = c(1900, 1340), f3 = 3160, f4 = 4430), rolloff = c(-7.5, -6)* ^§^	1,369	-1.8 dB for rising F2
Spectral centroid	Boosted vs. dampened high frequencies in source spectrum	Weak harmonics, dampened high frequencies: rolloff = -13	911	Strong harmonics, boosted high frequencies: rolloff = -3	2,170	-3.5 dB for high spectral centroid
Trill	Alveolar trill vs. no trill	~100 ms trill: [rə]^¶^	1,443	No trill: [ə]	1,601	-5.8 dB for no trill

§ The “rolloff” parameter controls source spectrum, and it was dynamically adjusted to keep the amount of high-frequency in the spectrum relatively stable, since otherwise changing the frequency of F1 or F2 would have changed the overall spectral slope

¶ The trill was synthesized using amplitude modulation, F4 transitions, and rolloff modulation

See R code in the Online Electronic Supplements for implementation details

### Procedure

We implemented a web-based html version of the implicit associations test (IAT) closely following the procedure described by Parise and Spence (2012). The task was to learn a rule associating the left arrow with one color and sound and the right arrow with another color and sound. Participants could examine the rule and hear the sounds for an unlimited amount of time before each block. For example, in one block of trials light gray/high pitch might be assigned to the left key and dark gray/low pitch to the right key. In the next block the rule would change, and all four possible combinations would recur in random order in multiple blocks throughout the experiment.

At the beginning of the experiment the participant was presented with instructions in the form of text and several slides followed by two blocks of 16 practice trials each. On the rare occasions when the accuracy was lower than the target level of 75%, practice blocks were repeated as many times as necessary. Once the participant had understood the procedure and achieved accuracy of 75% or better, they proceeded to complete 16 test blocks of 16 trials each.

As each trial began, a fixation cross was shown in the middle of the browser screen for a random period of 500–600 ms. After a delay of 300–400 ms the stimulus was presented. Color stimuli were shown for 400 ms in the same location as the fixation cross against a uniform white background; sounds also lasted about 400 ms. As soon as the stimulus disappeared or stopped playing, response buttons were activated and remained active until the participant had pressed the left/right arrows on the keyboard or clicked the corresponding buttons on the screen (the latter option was added for those participants who performed the experiment on a device without a physical keyboard). If the response was correct, the next trial began immediately. If it was incorrect, a red warning cross was flashed for 500 ms. Response arrows remained visible on the screen throughout the trials, but they were active only during the response phase. The experiment lasted between 10 and 30 min, depending primarily on how quickly the participant mastered the procedure.

The screens and speakers used by participants were not calibrated, and in general we had no control over the devices that were used in the online experiment. However, the main variable of interest in this experiment was within-subject difference in response time and accuracy depending on sound-color pairing. As such, it was not essential for us to standardize the absolute physical characteristics of the presented colors and sounds, but only to preserve the relevant contrasts between stimuli pairs.

### Participants

Participants were recruited via https://www.prolific.ac and reimbursed with £2–£2.5. They performed the study online, using a personal computer or a mobile device. All participants reported that they were fluent in English, had normal or corrected-to-normal vision, and had normal color perception. Submissions were discarded if they contained fewer than eight out of 16 complete blocks or if the average accuracy across all blocks was under 75%. A new sample of 20 participants was recruited for each of 22 experiments (N = 20 × 22 = 440 approved submissions, range 17–24 per experiment). Participants were not prevented from taking part in multiple experiments, so the total number of unique individuals across 22 experiments was 385 instead of 440. The mean number of completed test trials per participant was 253 out of 256.

### Statistical analysis

All practice trials were discarded, and only test trials were analyzed (*N* = 111,532 trials). We worked with unaggregated, trial-level data and fit mixed models with a random intercept per target stimulus and a random intercept and slope per subject. The main predictor of interest was the rule for color-sound association in the current block. For example, in the luminance-loudness experiment light gray could be associated with the loud or quiet sound and assigned to the left or right key, for a total of four possible rules. However, there was no obvious side bias in response patterns, reducing four rules to two conditions: (1) light = loud, dark = quiet, and (2) light = quiet, dark = loud. The random intercept per target primarily captured the variance in accuracy or response time (RT) depending on the modality of the stimulus (e.g., response to visual stimuli was considerably faster than to acoustic stimuli). The random intercept per participant was included to account for individual differences in both accuracy and RT, which also accounted for possible differences in RT due to the chosen method of responding (with the keyboard, touchscreen, or mouse). Finally, we allowed the effect of condition to vary across participants by including a random slope per subject. Model comparison with information criteria suggested that the random slope improved predictive accuracy only in those experiments in which the congruence effect was weak and highly variable across participants (details not shown). Nevertheless, we included the random slope in all models, so as to keep them consistent and to be able to estimate cross-modal correspondences for each individual participant.

Two Bayesian mixed models of the same structure were fit for each experiment: a logistic model predicting accuracy and a log-normal model predicting RT in correct trials. Both models were fit in a Stan computational framework (http://mc-stan.org/) accessed from R using a *brms* package (Bürkner, 2017). We specified mildly informative regularizing priors on regression coefficients so as to reduce overfitting and improve convergence. When analyzing RT, we excluded all trials with incorrect responses (on average ~5%, no more than 25% per participant according to exclusion criteria) or with RT over 5000 ms (~0.3% of trials). To improve transparency, in Table 4 we report both observed and fitted values from regression models.

**Table 4:** Error rates and response times in 22 separate experiments

Acoustic contrast	Visual contrast	Rule	Error rate, %			Response time, ms		
			Observed (mean)	Fitted	Difference [95% CI]	Observed (mean)	Fitted	Difference [95% CI]
Loudness	L	Loud = light gray	6.2	4.8	3.9 [1.1–13.6]	1,451	1,268	128 [63–211]
		Loud = dark gray	1.2	0.9		1,190	1,140	
	a	Loud = red	4.2	3.1	0.4 [-0.6–2.2]	1,196	1,129	34 [3–71]
		Loud = green	3.5	2.6		1,157	1,094	
	b	Loud = yellow	4.4	3.9	1.6 [0.2–3.9]	1,105	1,042	21 [-6–54]
		Loud = blue	3.4	2.2		1,056	1,020	
	Sat	Loud = unsaturated	7.4	6.5	4.1 [1.9–8.5]	1,223	1,145	84 [39–137]
		Loud = saturated	3	2.4		1,113	1,061	
Pitch	L	High pitch = dark gray	8.3	6	3.2 [0.4–14.3]	1,201	1,137	64 [16–121]
		High pitch = light gray	4.4	2.8		1,153	1,075	
	a	High pitch = green	3.4	2.4	-0.3 [-2.3–0.6]	1,196	1,118	-10 [-37–17]
		High pitch = red	3.9	2.8		1,211	1,127	
	b	High pitch = yellow	5.2	3.9	1.1 [0.0–3.5]	1,358	1,212	49 [10–96]
		High pitch = blue	4	2.8		1,268	1,161	
	Sat	High pitch = unsaturated	9.9	7	4.9 [1.6–13.2]	1,416	1,296	108 [59–177]
		High pitch = saturated	4.7	2.1		1,259	1,188	
F1	L	High F1 = dark gray	11.6	9.3	0.5 [-2.6–4.6]	1,200	1,118	6 [-18–33]
		High F1 = light gray	11.5	8.6		1,200	1,112	
	a	High F1 = green	6.1	4.2	-0.1 [-2.4–1.8]	1,203	1,134	-22 [-59–9]
		High F1 = red	6.5	4.3		1,221	1,157	
	b	High F1 = blue	5.3	4.4	-0.7 [-3.6–0.8]	1,219	1,128	-8 [-37–19]
		High F1 = yellow	6.3	5.2		1,221	1,137	
F2	L	High F2 = dark gray	5.8	4.2	0.2 [-1.5–2.0]	1,164	1,103	16 [-16–48]
		High F2 = light gray	5.3	4		1,159	1,087	
	a	High F2 = green	4.1	2.5	-0.8 [-3.0–0.6]	1,291	1,092	-21 [-49–5]
		High F2 = red	4.7	3.4		1,168	1,112	
	b	High F1 = blue	3.6	2.3	-0.2 [-1.3–0.8]	1,151	1,071	-29 [-70–9]
		High F1 = yellow	3.8	2.5		1,160	1,100	
Spectrum	L	High freq = dark gray	7.6	5.9	4.0 [1./0 12.5]	1,287	1,203	83 [30–148]
		High freq = light gray	3	1.8		1,159	1,119	
	a	High freq = green	6.6	3.6	0.4 [-1.5–3.0]	1,017	959	-18 [-49–8]
		High freq = red	6.5	3.1		1,036	977	
	b	High freq = yellow	7.7	5.7	1.5 [-0.1–4.5]	1,169	1,114	25 [-3–59]
		High freq = blue	5.6	4.1		1,163	1,088	
	Sat	High freq = unsaturated	9.1	6.8	3.5 [0.5–10]	1,342	1,217	55 [9–109]
		High freq = saturated	6.1	3.1		1,261	1,163	
Trill	L	Trill = light gray	7	4.5	2.5 [0.6–8.2]	1,389	1,258	82 [34–146]
		Trill = dark gray	4.4	1.9		1,266	1,175	
	a	Trill = green	4.6	3.1	1.0 [-0.4–6.3]	1,111	1,052	9 [-15–32]
		Trill = red	3.3	1.9		1,100	1,043	
	b	Trill = blue	3.4	2.3	1.1 [0.2–4.6]	1,167	1,090	22 [-8–61]
		Trill = yellow	2.2	1.1		1,143	1,067	
	Sat	Trill = saturated	5.7	3.3	1.2 [-0.4–4.4]	1,244	1,183	43 [1–92]

## Results

The accuracy and speed of responding across all 22 experiments are summarized in Table 4. Accuracy was generally high, with the average error rate between 1% and 11% across experiments. RT in trials with a correct response was on average about 900–1,200 ms, which is slower than reported by Parise and Spence (2012). Since participants were instructed to achieve at least 75% accuracy, some may have prioritized avoiding mistakes at the cost of slowing down. In general, there is a trade-off between accuracy and speed in the IAT: some participants reveal their implicit associations by making more mistakes in the incongruent condition, while others maintain high accuracy but take longer to respond. We therefore looked for the effect of sound-color pairing on both accuracy and RT (Table 4). When both models showed significant differences in the same direction (i.e., both more errors and longer RT in condition 1 than in condition 2), that provided particularly clear evidence of non-arbitrary sound-color associations.

**Fig. 1:**
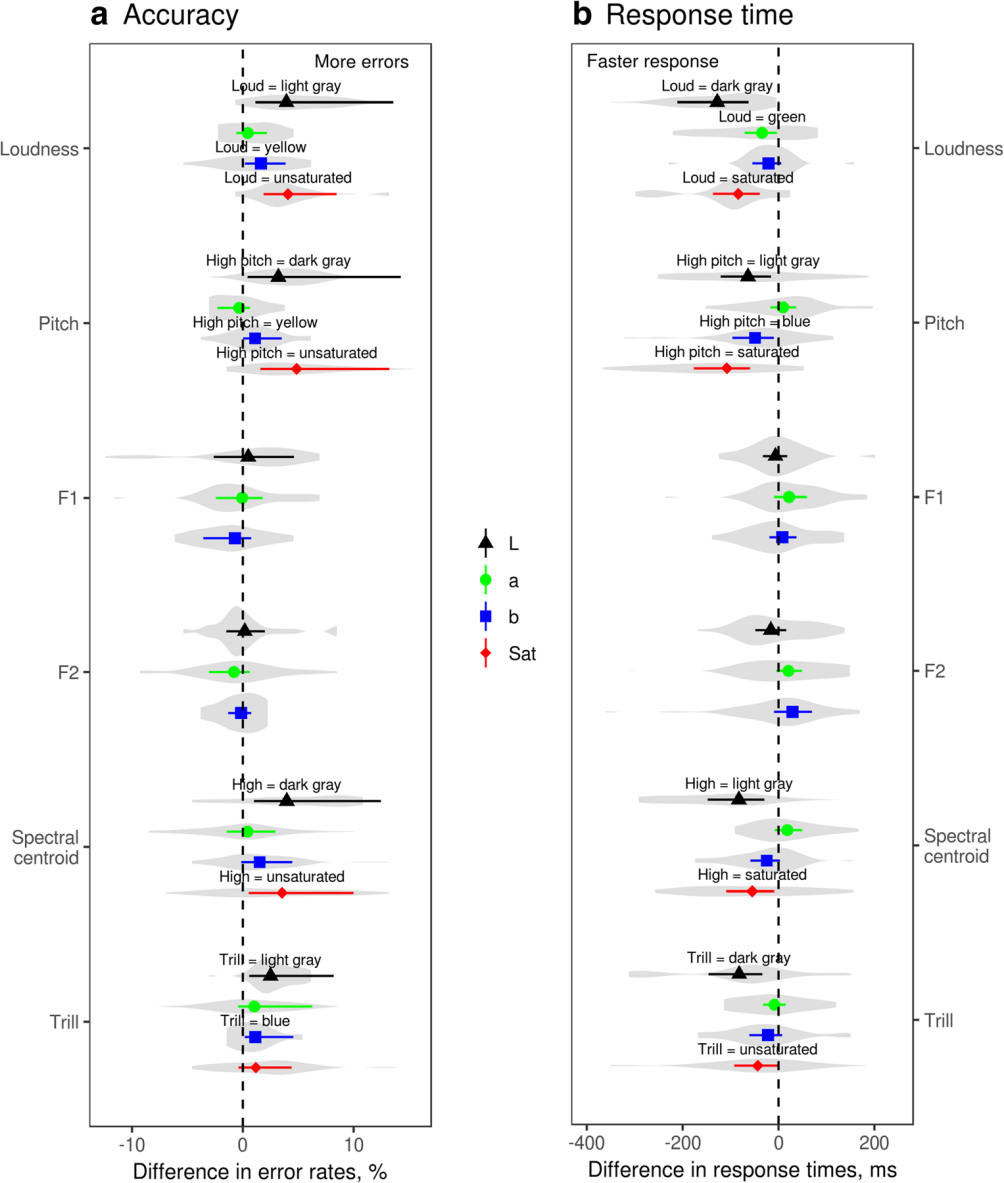
Predicted difference in error rates (**A**) and response times (**B**) depending on the rule for pairing sounds and colors in 22 separate experiments. Solid points and error bars show the median of the posterior distribution and 95% CI. Labeled points have confidence intervals that do not overlap with zero. Violin plots show the distribution of observed values of the contrasts across participants (~20 per experiment, *N* = 440). *L* = luminance, *a* = green-red, *b* = yellow-blue, *Sat* = saturation

The findings are summarized graphically in Fig. 1, which also shows the distribution of average contrasts across participants. Higher luminance (light vs. dark gray on white background) was associated with lower loudness, higher pitch, higher spectral centroid, and the presence of a trill. The effect size for luminance was 3–4% difference in error rates and 60–120 ms difference in RT (Table 4). Congruency effects were revealed by both accuracy and RT, and were in the same direction for most participants. In contrast, there was no association between luminance and F1 or F2 frequency.

Neither green-red nor yellow-blue hue contrasts were reliably associated with any of the tested acoustic features, with one exception: high pitch was associated with blue (vs. yellow) hue (Table 4, Fig. 1). This effect was relatively small, but its confidence intervals excluded zero for both error rates (1.1% fewer errors, 95% CI 0–3.5) and response time (49 ms, 95% CI 10–96). In addition, a statistically marginal, but logically consistent congruence effect was observed between high spectral centroid and blue (vs. yellow) hue, again for both error rates (1.5%, 95% CI -0.1–4.5) and RTs (25 ms, 95% CI -3–59). The effect size for hue contrasts (0–1.5% and 0–50 ms) was thus about half of that for luminance contrasts. A few more marginal effects for hue-sound associations are shown in Fig. 1, but all of them were weak and manifested either in error rates or response times, but not both. We therefore do not consider them further.

Finally, high (vs. low) saturation was associated with greater loudness, higher pitch, and higher spectral centroid. In addition, the sound with a trill was weakly associated with low saturation based on the response time (43 ms, 95% CI 1–92), but only marginally so based on error rates (1.2%, 95% CI -0.4–4.4).

## Discussion

In a series of experiments we used the implicit associations test (IAT) to investigate cross-modal correspondences between separately manipulated visual and acoustic features. This work extends previous research in two important ways. First, the majority of earlier studies relied on explicit matching, which quickly generates large amounts of data but operates at the relatively high “decisional level” (Spence, 2011) of consciously available beliefs. In contrast, implicit tasks like the IAT require more data, but they offer an insight into lower-level processing of perceptual input and thus provide a useful complementary perspective on sound-color associations. Second, we aimed to further refine the control over both visual and acoustic features, building upon several recent studies that employed perceptually accurate color spaces and sophisticated methods of sound synthesis (Hamilton-Fletcher et al., 2017; Kim et al., 2017). We created complex, vowel-like acoustic stimuli with a formant synthesizer, combining natural-sounding voice quality with precise control over formants, spectral envelope, intonation, loudness, and amplitude modulation. This enabled us to explore novel acoustic features in synthetic vowels, notably formant frequencies and spectral centroid, while avoiding several potential acoustic confounds. Visual stimuli were created using the *Lab* color space and varied along one dimension at a time (luminance, hue, or saturation). This experimental technique has the potential to pinpoint the individual visual and acoustic features driving cross-modal correspondences at a perceptual level. At the same time, the methodological differences between the current project and most previous research, particularly the use of an implicit outcome measure and complex, vowel-like sounds instead of pure tones, call for caution when directly comparing the results. In many cases our data confirm or nuance previous observations, but there are also several important differences, as discussed below.

In this study light gray was associated with low loudness and dark gray with high loudness, which seemingly contradicts the often reported association of visual luminance with auditory loudness (Table 1). However, the context in which stimuli varying in luminance are presented may strongly affect the result. The brightness of a physical source of light, such as a light bulb, seems to be unequivocally mapped onto the loudness of an accompanying sound (Bond & Stevens, 1969; Root & Ross, 1965). When the visual stimuli are patches of color, however, the way their lightness is mapped onto loudness depends on the background (Hubbard, 1996; Marks, 1974, 1987). When the background is darker than both stimuli, lighter colors are associated with louder sounds. When the background is intermediate in luminance between that of the stimuli, the association becomes inconsistent (Marks, 1974, 1987), unless the background is more similar in luminance to one stimulus than to the other (e.g., in Martino & Marks, 1999). The likely explanation is that luminance-loudness associations are driven by the amount of contrast between the stimulus and the background – more generally, by visual saliency (Itti & Koch, 2000) – rather than by lightness or luminance as such. In our experiment, visual stimuli (dark gray and light gray squares) were presented against a white background, making the dark stimulus more salient and therefore causing it to be associated with the louder of two sounds. It is also worth pointing out that the same effect was observed consistently for practically all participants (Fig. 1).

Interestingly, higher pitch was associated with light as opposed to dark gray, even though the association of dark gray with loudness indicates that the dark stimulus had higher visual saliency. This dissociation between pitch and loudness suggests that two different mechanisms are responsible for cross-modal correspondences between luminance and loudness, on the one hand, and luminance and pitch, on the other. We suggest that the luminance-loudness associations are prothetic (quantitative) in nature and driven by congruence in visual and auditory saliency, making them sensitive to contextual effects such as background color. In contrast, luminance-pitch appears to be a metathetic (qualitative) cross-modal correspondence. The same pattern was observed when both sounds had the same pitch and differed only in their spectral centroid: the sound with stronger upper harmonics and thus higher spectral centroid was associated with light versus dark gray. This is a novel finding in the context of research on sound-color associations, but it is fully in accord with the well-established fact that human ratings of timbral brightness or sharpness correlate closely with spectral centroid (Fastl & Zwicker, 2006; Schubert et al., 2004). We can thus conclude that lighter colors are mapped not only onto a higher pitch, but also onto an upward shift in spectral energy, even without a change in the fundamental frequency. This has important consequences for the likely interpretation of associations between formant frequencies and colors (see below). It is also worth reiterating that in our study the association between auditory frequency and luminance was not mediated by differences in perceived loudness since we normalized the stimuli for subjective loudness (as also reported by Hamilton-Fletcher et al., 2017).

Unlike luminance, saturation displayed the same pattern of association with loudness (loud = saturated) and with auditory frequency (high pitch or high spectral centroid = saturated). Hamilton-Fletcher and co-authors (Hamilton-Fletcher et al., 2017) suggest that the association between saturation and several acoustic characteristics – such as loudness, pitch, and spectral centroid – is based on ranking stimuli along each dimension from low to high, and therefore in essence these are prothetic cross-modal correspondences. This explanation is consistent with our results for saturation, since it was indeed associated with higher loudness, pitch, and spectral centroid, but this logic breaks down when applied to luminance. Since we established that the dark gray stimulus was the marked, more salient visual stimulus, we would expect dark gray to be paired with higher pitch if this association was prothetic. In actual fact, however, higher pitch was associated with a lighter (in this case less salient) color, as was also reported in numerous earlier studies (Table 1). One explanation is that auditory frequency can be compared to other modalities either qualitatively (higher frequency = lighter color) or quantitatively (“more” frequency = “more” saturation), perhaps depending on the existence and strength of pre-existing cross-modal correspondences. For example, if there is a powerful metathetic association of high frequency with lighter colors, it might override the weaker prothetic alignment of low-to-high visual saliency (which in this case was the reverse of lightness) with low-to-high frequency. Other explanations are certainly possible, and the exact cognitive mechanisms responsible for the observed cross-modal correspondences are yet to be elucidated.

Moving on to other findings, we did not observe any association between changes in the frequencies of the first two formants and either luminance or hue of the presented colors. We did not test for an association between formants and saturation, but it appears unlikely that there would be any. This null result contradicts a rich research tradition (Marks, 1975), according to which most informants agree which vowels best match which colors. However, natural focal colors differ not only in hue, but also in luminance and saturation. In more recent experimental research there have been attempts to use multiple regression (Moos et al., 2014) or palettes of equiluminant colors (Hamilton-Fletcher et al., 2017) to tease apart the contributions of these color dimensions, but even these better controlled studies did not distinguish between formant frequencies and the overall distribution of spectral energy. An increase in formant frequency not only modifies vowel quality, but also strongly shifts the spectral centroid upwards, which is in itself sufficient to make a sound “brighter” (Fastl & Zwicker, 2006; Stevens, 2000). We dynamically adjusted the spectrum of our synthetic vowels, largely – but not completely – eliminating the effect of formant transitions on the overall distribution of energy in the spectrum. The resulting diphthongs were easily distinguishable by listeners, as evidenced by the high accuracy in the IAT, but the relatively stable spectral centroid prevented the sounds with higher formants from sounding “brighter,” canceling out an otherwise expected association between higher formants and higher luminance. Since we also demonstrated a clear association between spectral centroid and luminance, the logical conclusion seems to be that the often reported associations between formants and luminance are driven by the spectral consequences of formant transitions in natural vowels, not by formant frequencies per se. In other words, perceptually “bright” vowels, such as [i] and [a] (Johansson, Anikin, & Aseyev, 2018), probably owe their brightness to the fact that raising the frequency of individual formants (F2 for [i], F1 for [a]) shifts the balance of low- and high-frequency energy in the spectrum. If that is true, it should be possible to manipulate the perceived “brightness” of any vowel without changing its nature, simply by boosting or dampening higher frequencies in the spectrum, which can be verified in future studies.

One of the most surprising findings was the nearly complete lack of association between hue and any of the tested acoustic contrasts, with the possible exception of the relatively weak tendency to match higher pitch and higher spectral centroid with blue (vs. yellow) hue. It is possible that the effect size for hue was too small, falling below the sensitivity threshold of the experimental method. Alternatively, the previously reported hue-sound associations may only manifest themselves in the context of explicit matching. There is a considerable body of evidence, including a few studies that controlled for luminance (Hamilton-Fletcher et al., 2017; Moos et al., 2014; Kim et al., 2017), proving that informants consistently match hue to pitch, loudness, and formant frequencies. On the other hand, the weak IAT results suggest that hue may be associated with sound on a higher conceptual level through a mechanism that we tentatively labeled “semantic matching” in Table 1. For example, participants faced with a range of equiluminant colors might match high-frequency sounds with yellowish hues (Hamilton-Fletcher et al., 2017) by means of re-categorizing the available hues in terms of lexically labeled focal colors, so that the presented “yellowish” hue is treated as an approximation to the focal yellow, which would indeed be the best match due to its superior brightness. In an implicit task, however, this association disappears or can even be reversed, so that high pitch matches blue instead of yellow, as in the present study. Likewise, listeners may have relatively stable internal representations of different vowel sounds, so that [u] might be perceived and explicitly classified as “dark” and [i] as “bright” even if the stimuli are acoustically filtered, giving the [u] more high-frequency spectral energy. Although post-perceptual cross-modal correspondences have been observed with the IAT (Lacey et al., 2016), high-level, non-automatic, and relatively slow effects of this kind may manifest themselves more readily in explicit as opposed to implicit tests. This explanation is highly speculative, and our results will need to be replicated. But even with these provisos, the present findings clearly show that prothetic, low-to-high dimensions of color – luminance and saturation – dominate over hue in the context of implicit cross-modal matching.

The most acoustically complicated manipulation in the present study was to add rapid, trill-like amplitude modulation at the beginning of a syllable, leaving the other stimulus in the pair without a trill. While interesting from a linguistic point of view, this manipulation is difficult to interpret because it introduces two acoustic contrasts instead of one. The syllable with a trill is marked by virtue of containing an additional phoneme, but it also has a noticeably lower spectral centroid (Table 3). Listeners associated the trill with dark (vs. light) gray and, marginally, with low (vs. high) saturation. The association with luminance may be a case of prothetic matching of visual saliency (higher for dark gray) and acoustic saliency (higher for the marked syllable with a trill). Alternatively, this effect may be mediated by an association between spectral centroid (higher without a trill) and color lightness, which would also explain why the trill was associated with low rather than high saturation. Both of these effects may also be present simultaneously; in fact, summation of cross-modal correspondences has been shown experimentally (Jonas et al., 2017), and it may be a common occurrence in the real world, where objects have more than two sensory dimensions. This ambiguity showcases one of the problems facing cross-modal research, namely the inevitable tradeoff between the control over experimental stimuli and their ecological validity. It is also worth pointing out that, in contrast to some previous results (Johansson, Anikin, Carling, et al., 2018), we found no direct association between the trill and green-red contrast. On the other hand, linguistic studies of sound symbolism concern focal colors, which were not featured in the present study. Assuming that cool colors, such as blues and greens, are lower than warm colors in luminance and saturation, the presence of trills in words for the color green might still be sound symbolically charged, but this will have to be verified in future studies.

The study presented here has a number of other limitations. First of all, the chosen method of implicit associations required such a large sample that only a single pair of visual and acoustic stimuli could be tested within each condition. For example, “luminance” in the discussion above corresponds to the contrast between two shades of gray on the same white background, “pitch” represents a single, rather arbitrarily chosen contrast of six semitones, and so on. It remains to be seen how our conclusions will hold once a more diverse range of stimuli has been tested. Furthermore, online data collection entails certain methodological complications. For example, response times were on average about 1 s, which is slightly slower than in the study whose design we closely reproduced (Parise & Spence, 2012). One likely reason is that participants responded more slowly to the acoustic stimuli, which lasted 400 ms and in some conditions contained dynamic cues such as moving formants, making it necessary to hear the entire stimulus before even beginning to classify it. It is also possible that some participants were slowed down by using the mouse to click the response buttons instead of pressing keys on a physical keyboard or touching the buttons directly on the screen. An inability to standardize the equipment used by participants is one of the shortcomings of the present study, even though we could largely account for such variation by using a within-subject design and mixed models with a participant-specific intercept. A within-subject design is in general recommended in the context of online research, particularly when the outcome measure is device-dependent, as in the case of response time (Woods et al., 2015). Nevertheless, assuming that fast responses are relatively automatic, while slower responses are indicative of more extensive cognitive processing (Parise & Spence, 2012), it would be useful to replicate our results in a more controlled setting, ensuring that all participants pressed physical buttons and had less time for deliberation. This should make the estimates more precise and possibly reveal weaker cross-modal correspondences, for example, between loudness and hue or pitch and hue.

Taking a step back, the present method allowed us to study the interaction between perception, language, and cognition by isolating relevant visual and acoustic parameters without disconnecting them too much from natural speech sounds and the colors we perceive in the surrounding world. An important avenue for further research is to investigate how the discovered perceptual sound-color associations relate to sound symbolism in names of colors in natural languages. The mapping of high pitch and high spectral centroid on lighter colors is largely in line with previous cross-linguistic studies that have shown associations between [u] and concepts denoting darkness (Blasi et al., 2016). In a follow-up study (Johansson, Anikin, et al., 2018) we confirmed that both sonorous and bright vowels are strongly over-represented in the names of bright colors across world languages, while sonorous consonants are over-represented in the names of saturated colors. Interestingly, in the present study we observed implicit cross-modal correspondences for spectral centroid, but not formant frequencies (which define vowel quality), confirming that sound symbolism operates at the level of individual acoustic features rather than phonemes (Sidhu & Pexman, 2018). Together with other evidence of cross-modal correspondences on a basic perceptual level (Hamilton-Fletcher et al., 2017; Kim et al., 2017), the present findings also indicate that sound-meaning associations do not have to be mediated by orthography (cf. Nielsen & Rendall, 2011). A similar experimental approach can be useful for research on other audiovisual correspondences beyond the domain of color (Walker, 2012) as well as for research on other sensory modalities. Likewise, the differences between prothetic and metathetic mappings, as well as the fact that luminance and saturation were found to be the driving factors in sound-color mappings, add a further dimension to our understanding of how iconic associations are grounded and operate on semantic, phonetic, semiotic, and cognitive levels. Crucially, luminance, followed by saturation and the possible association of cool colors and trills, emerges as the primary visual component in color-sound symbolism, although its role should be further investigated in words of natural languages in order to connect cross-modal correspondences on a perceptual level with the development and change of lexicalization patterns and semantic boundaries across languages.

## Conclusions

Using the implicit associations test, we confirmed the following previously reported cross-modal correspondences between visual and acoustic features:

- high loudness with high saturation,
- high pitch with high luminance,
- high pitch with high saturation,
- high spectral centroid with high saturation.

We propose to reinterpret the following associations:

- loudness with luminance: driven by visual saliency rather than color lightness, therefore reversed when more luminant stimuli are less salient,
- high formants with high luminance and saturation: driven by spectral shape rather than vowel quality, therefore no effect when controlling for spectral centroid.

We also report two purportedly novel associations:

- high spectral centroid with high luminance,
- alveolar trill with low luminance and low saturation.

Finally, none of the previously reported associations between hue and acoustic features were observed in the IAT, with the possible exception of a marginal and previously unreported tendency to match high pitch with blue (vs. yellow) hue.

## References

[CR1] Anikin, A. (2018). Soundgen: An open-source tool for synthesizing nonverbal vocalizations. *Behavoir Research Methods*, 1-15. 10.3758/s13428-018-1095-7 10.3758/s13428-018-1095-7 PMC6478631 30054898

[CR2] Bankieris K, Simner J (2015). What is the link between synaesthesia and sound symbolism?. Cognition.

[CR3] Bernstein IH, Edelstein BA (1971). Effects of some variations in auditory input upon visual choice reaction time. Journal of Experimental Psychology.

[CR4] Blasi DE, Wichmann S, Hammarström H, Stadler PF, Christiansen MH (2016). Sound–meaning association biases evidenced across thousands of languages. Proceedings of the National Academy of Sciences.

[CR5] Bond B, Stevens SS (1969). Cross-modality matching of brightness to loudness by 5-year-olds. Perception & Psychophysics.

[CR6] Bürkner, P. C. (2017). brms: An R package for Bayesian multilevel models using Stan. *Journal of Statistical Software, 80*(1), 1-28.

[CR7] Evans, K. K, & Treisman, A. (2010). Natural cross-modal mappings between visual and auditory features. *Journal of Vision, 10*(1):6, 1-12. 10.1167/10.1.6 PMC2920420 20143899

[CR8] Fastl H, Zwicker E (2006). *Psychoacoustics: Facts and models, 2*^*nd*^*ed*.

[CR9] Giannakis, K. (2001). Sound mosaics: A graphical user interface for sound synthesis based on audio-visual associations. Doctoral dissertation, Middlesex University, UK. Retrieved from http://eprints.mdx.ac.uk/6634/1/Konstantinos-sound_mosaics.phd.pdf

[CR10] Hamilton-Fletcher G, Witzel C, Reby D, Ward J (2017). Sound properties associated with equiluminant colours. Multisensory Research.

[CR11] Hubbard TL (1996). Synesthesia-like mappings of lightness, pitch, and melodic interval. The American Journal of Psychology.

[CR12] Itti L, Koch C (2000). A saliency-based search mechanism for overt and covert shifts of visual attention. Vision Research.

[CR13] Jakobson R (1962). *Selected writings I. Phonological studies*.

[CR14] Johansson, N., Anikin, A., & Aseyev, N. (2018). *Color-sound symbolism in natural languages.* Manuscript in preparation.

[CR15] Johansson, N., Anikin, A., Carling, G., & Holmer, A. (2018). *The typology of sound symbolism: Defining macro-concepts via their semantic and phonetic features*. Manuscript submitted for publication.

[CR16] Jonas C, Spiller MJ, Hibbard P (2017). Summation of visual attributes in auditory–visual crossmodal correspondences. Psychonomic Bulletin & Review.

[CR17] Kim HW, Nam H, Kim CY (2017). [i] is lighter and more greenish than [o]: Intrinsic association between vowel sounds and colors. Multisensory Research.

[CR18] Kim, K. H., Gejima, A., Iwamiya, S. I., & Takada, M. (2011). The effect of chroma of color on perceived loudness caused by noise. In *40th International Congress and Exposition on Noise Control Engineering 2011, 4* (pp. 3151–3156).

[CR19] Klapetek A, Ngo MK, Spence C (2012). Does crossmodal correspondence modulate the facilitatory effect of auditory cues on visual search?. Attention, Perception, & Psychophysics.

[CR20] Lacey S, Martinez M, McCormick K, Sathian K (2016). Synesthesia strengthens sound-symbolic cross modal correspondences. European Journal of Neuroscience.

[CR21] Ladefoged P, Maddieson I (1996). *The sounds of the world's languages*.

[CR22] Ludwig VU, Adachi I, Matsuzawa T (2011). Visuoauditory mappings between high luminance and high pitch are shared by chimpanzees (Pan troglodytes) and humans. PNAS.

[CR23] Marks LE (1974). On associations of light and sound: The mediation of brightness, pitch, and loudness. The American Journal of Psychology.

[CR24] Marks LE (1975). On colored-hearing synesthesia: Cross-modal translations of sensory dimensions. Psychological Bulletin.

[CR25] Marks LE (1987). On cross-modal similarity: Auditory–visual interactions in speeded discrimination. Journal of Experimental Psychology: Human Perception and Performance.

[CR26] Martino G, Marks LE (1999). Perceptual and linguistic interactions in speeded classification: Tests of the semantic coding hypothesis. Perception.

[CR27] Melara RD (1989). Dimensional interaction between color and pitch. Journal of Experimental Psychology: Human Perception and Performance.

[CR28] Menzel, D., Haufe, N., & Fastl, H. (2010). Colour-influences on loudness judgements. In *Proc. 20th Intern. Congress on Acoustics, ICA (2010), Sydney, Australia*.

[CR29] Mielke, J. (2004–2018). *P-base. A database of phonological patterns*. http://pbase.phon.chass.ncsu.edu.

[CR30] Miyahara T, Koda A, Sekiguchi R, Amemiya T (2012). A psychological experiment on the correspondence between colors and voiced vowels in non-synesthetes. Kansei Engineering International Journal.

[CR31] Mondloch CJ, Maurer D (2004). Do small white balls squeak? Pitch-object correspondences in young children. Cognitive, Affective, & Behavioral Neuroscience.

[CR32] Moos A, Smith R, Miller SR, Simmons DR (2014). Cross-modal associations in synaesthesia: Vowel colours in the ear of the beholder. i-Perception.

[CR33] Moran, S., McCloy, D. & Wright, R. (eds.) (2014). *PHOIBLE Online*. Leipzig: Max Planck Institute for Evolutionary Anthropology. http://phoible.org.

[CR34] Nielsen A, Rendall D (2011). The sound of round: Evaluating the sound-symbolic role of consonants in the classic Takete-Maluma phenomenon. Canadian Journal of Experimental Psychology/Revue canadienne de psychologie expérimentale.

[CR35] Orlandatou, K. (2012). The role of pitch and timbre in the synaesthetic experience. In *Proceedings of the 12th International Conference on Music Perception and Cognition and the 8th Triennial Conference of the European Society for the Cognitive Sciences of Music, Thessaloniki, Greece* (pp. 751-758).

[CR36] Panek W, Stevens SS (1966). Saturation of red: A prothetic continuum. Perception & Psychophysics.

[CR37] Parise CV, Spence C (2012). Audiovisual crossmodal correspondences and sound symbolism: A study using the implicit association test. Experimental Brain Research.

[CR38] Ramachandran VS, Hubbard EM (2001). Synaesthesia – A window into perception, thought and language. Journal of Consciousness Studies.

[CR39] Root RT, Ross S (1965). Further validation of subjective scales for loudness and brightness by means of cross-modality matching. The American Journal of Psychology.

[CR40] Schubert, E., Wolfe, J., & Tarnopolsky, A. (2004). Spectral centroid and timbre in complex, multiple instrumental textures. In *Proceedings of the international conference on music perception and cognition, North Western University, Illinois* (pp. 112-116).

[CR41] Sidhu DM, Pexman PM (2018). Five mechanisms of sound symbolic association. Psychonomic Bulletin & Review.

[CR42] Simpson RH, Quinn M, Ausubel DP (1956). Synesthesia in children: Association of colors with pure tone frequencies. The Journal of Genetic Psychology.

[CR43] Spence C (2011). Crossmodal correspondences: A tutorial review. Attention, Perception, & Psychophysics.

[CR44] Stevens KN (2000). *Acoustic phonetics*.

[CR45] Walker P (2012). Cross-sensory correspondences and cross talk between dimensions of connotative meaning: Visual angularity is hard, high-pitched, and bright. Attention, Perception, & Psychophysics.

[CR46] Ward J (2013). Synesthesia. Annual Review of Psychology.

[CR47] Ward J, Huckstep B, Tsakanikos E (2006). Sound-colour synaesthesia: To what extent does it use cross-modal mechanisms common to us all?. Cortex.

[CR48] Watanabe, K., Greenberg, Y., & Sagisaka, Y. (2014). Sentiment analysis of color attributes derived from vowel sound impression for multimodal expression. In *Signal and Information Processing Association Annual Summit and Conference (APSIPA), 2014 Asia-Pacific* (pp. 1-5).

[CR49] Witzel C, Franklin A (2014). Do focal colors look particularly “colorful”?. JOSA A.

[CR50] Woods AT, Velasco C, Levitan CA, Wan X, Spence C (2015). Conducting perception research over the internet: a tutorial review. PeerJ.

[CR51] Wrembel M (2009). On hearing colours—cross-modal associations in vowel perception in a non-synaesthetic population. Poznań Studies in Contemporary Linguistics.

